# Depletion of NEAT1 lncRNA attenuates nucleolar stress by releasing sequestered P54nrb and PSF to facilitate c-Myc translation

**DOI:** 10.1371/journal.pone.0173494

**Published:** 2017-03-13

**Authors:** Wen Shen, Xue-hai Liang, Hong Sun, Cheryl L. De Hoyos, Stanley T. Crooke

**Affiliations:** Department of Core Antisense Research, IONIS Pharmaceuticals, Inc. 2855 Gazelle Court, Carlsbad, CA, United States of America; Medical Faculty Mannheim, University of Heidelberg, GERMANY

## Abstract

Altered expression of NEAT1, the architectural long non-coding RNA (lncRNA) of nuclear paraspeckles, has been reported during tumorigenesis, as well as under various cellular stress conditions. Here we report that the depletion of NEAT1 lncRNA alleviates nucleolar stress during RNAP I inhibition through releasing sequestered P54nrb and PSF to facilitate the IRES-dependent translation of c-Myc. RNAP I inhibitor CX5461 disrupts the SL1-rDNA interaction and induces nucleolar disruption, demonstrated by the accumulation of fibrillarin-containing nucleoplasmic foci and nucleolar clearance of ribosomal proteins in HeLa cells. Antisense oligonucleotide-mediated depletion of NEAT1 lncRNA significantly attenuated the RNAP I inhibition and its related nucleolar disruption. Interestingly, induction in the levels of c-Myc protein was observed in NEAT1-depeleted cells under RNAP I inhibition. NEAT1-associated paraspeckle proteins P54nrb and PSF have been reported as positive regulators of c-Myc translation through interaction with c-Myc IRES. Indeed, an increased association of P54nrb and PSF with c-Myc mRNA was observed in NEAT1-depleted cells. Moreover, apoptosis was observed in HeLa cells depleted of P54nrb and PSF, further confirming the positive involvement of P54nrb and PSF in cell proliferation. Together, our results suggest that NEAT1 depletion rescues CX5461-induced nucleolar stress through facilitating c-Myc translation by relocating P54nrb/PSF from nuclear paraspeckles to c-Myc mRNAs.

## Introduction

Paraspeckles are mammalian-specific RNA-protein structures with more than forty identified protein components [1]. Paraspeckles assemble hierarchically through the recruitment of protein components to the central scaffolding long non-coding RNA, NEAT1. As a result, NEAT1 RNA is indispensable to the maintenance of paraspeckle integrity. Complete disappearance of paraspeckles was reported in NEAT1^-/-^ MEF, while the exogenous expression of mouse NEAT1_2 in NEAT1^-/-^ MEF rescued paraspeckle formation [2]. Moreover, paraspeckles are absent in embryonic stem cells due to the lack of NEAT1 expression, but are formed immediately upon differentiation following the expression of NEAT1 [3]. Interestingly, this phenotype was one of the earlier lines of evidence showing that cell metabolism, differentiation in this case, can alter the abundance of NEAT1 RNA and the formation of paraspeckles.

Indeed, although no physiological defects under normal growth conditions were observed for NEAT1 knockout mice, levels of NEAT1 RNA and/or formation of paraspeckles were tightly coupled with various cellular stress conditions and changes in cell metabolism. For example, increasing in levels of NEAT1 lncRNA were observed upon immune stimuli [4], resulting in the excess formation of paraspeckles. In addition, p53-dependent induction of NEAT1 upon DNA damage was reported [5]. Moreover, NEAT1 upregulation also marks tumorigenesis and metastasis. For example, elevated levels of NEAT1 RNA and paraspeckle protein P54nrb were found in human breast cancer [6]. NEAT1 was also found to positively regulate the progression of prostate cancer by turning on transcription of prostate cancer related genes epigenetically [7].

Due to its architectural role in paraspeckle assembly, it is possible that NEAT1 is regulated in response to environmental or biochemical changes to rapidly sequester or release critical proteins to activate or suppress certain cellular processes. It has been reported that upon virus infection, levels of NEAT1 were induced to sequester PSF, the transcriptional repressor of an antiviral gene interleukin-8, activating the innate immune response against viral infection [4]. Most of the identified paraspeckle proteins have important functions in various cellular processes, including RNA synthesis, transcription regulation, apoptosis (*e*.*g*. RUNX3 and XIAP), proliferation (*e*.*g*. v-Fos), and neural development (*e*.*g*. FAM53A and FAM53B).

From proteins that associate with NEAT1, P54nrb and PSF are especially interesting. Protein levels of P54nrb and PSF correlate tightly with cell survival. It has been reported previously that P54nrb can protect neuron against neuronal apoptosis [8]. Oxidative stress-induced apoptosis was blocked in cells with exogenous expression of P54nrb. P54nrb also contributes to the insensitivity of melanoma cells against apoptosis [9]. Importantly, in agreement with their involvements in cellular proliferation, P54nrb and PSF were identified as positive regulators of translation of the proto-oncogene c-Myc [10]. Both proteins associate with a structural element located in the 5' untranslated region of the c-Myc mRNA termed an internal ribosome entry segment (IRES) and facilitate the cap-independent translation of c-Myc, especially during stress conditions.

Here we report that the depletion of NEAT1 RNA can alleviate nucleolar stress induced by the inhibition of rRNA synthesis in HeLa cells. During RNAP I inhibition, protein levels of c-Myc significantly increased in HeLa cells depleted of NEAT1 lncRNA. We further observed an increased association of P54nrb/PSF with c-Myc mRNA upon NEAT1 depletion, suggesting that the delocalization of P54nrb and PSF from nuclear paraspeckles to c-Myc mRNA occurred in the absence of NEAT1 during stress conditions. It has been well-studied that the levels of c-Myc protein positively correlate with RNAP I transcription. As a result, increasing in levels of c-Myc proteins in the absence of NEAT1 rescued rRNA transcription and nucleolar morphology during RNAP I inhibition.

## Materials and methods

### Cell culture and transfection

HeLa and MCF7 cells were grown at 37°C, 8% CO2 in DMEM supplemented with 10% FBS and 1% penicillin/streptomycin. Cells at 70% confluency were transfected with ASOs at specified concentration using lipofectamine 2000 (Thermo Fisher Scientific) at a final concentration of 4 μg/ml. siRNAs and ASOs are listed in S1 Text.

### RNA preparation and qRT-PCR

Total RNA was isolated using RNeasy Mini Kit (QIAGEN). One-step qRT-PCR was performed using QuantiTect Probe RT-PCR Kit (QIAGEN). Expression levels of target RNA were normalized to total RNA measured by Quant-iT RiboGreen RNA Reagent (Thermo Fisher Scientific). Expression levels of 47S rRNA precursor were examined using EXPRESS One-Step SYBR GreenER Kit (Thermo Fisher Scientific). Primer probe sets are listed in S1 Text.

### Western blotting

Western blotting was performed as described previously [11]. Antibodies for western blotting are listed in S1 Text.

### RNA-FISH and IF

RNA-FISH and IF were performed as described previously [11]. For more details, see the Supplemental Experimental Procedures. Antibodies for IF are listed in S1 Text.

### RNA-IP

RNA-IP was performed using Magna RIP Kit (Millipore) according to manufacturer’s instructions. Both input and co-immunoprecipitated RNAs were prepared by RNeasy Mini Kit (QIAGEN) and subjected to qRT-PCR analysis. Antibodies for RNA-IP are listed in S1 Text.

### Polysome profile analysis

Polysome profile analysis was performed using sucrose gradient fractionation as described previously [12].

## Results

### NEAT1-depletion rescued rRNA transcription in the presence of RNAP I inhibitor CX5461

**Fig 1:**
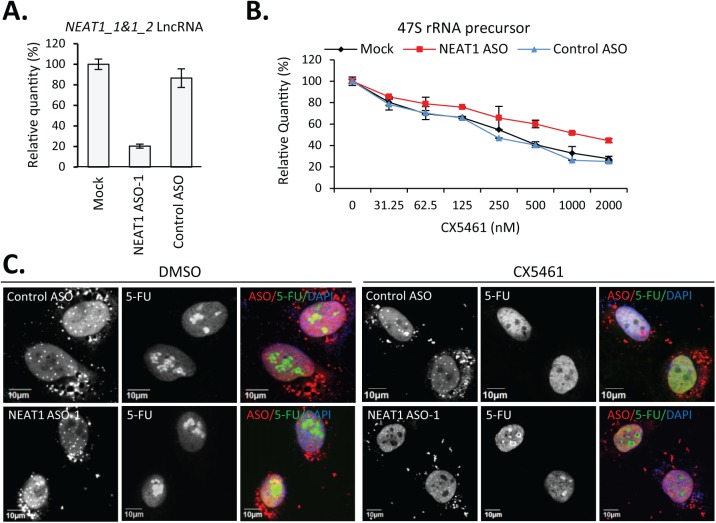
ASO-mediated depletion of NEAT1 partially rescued CX5461-induced transcriptional inhibition. **A.** qRT-PCR of the levels of NEAT1 lncRNA in HeLa cells either mock-treated or transfected with NEAT1 or control ASOs. **B.** Dose-dependent reduction of 47S rRNA precursor by CX5461 was partially rescued in NEAT1-depeted cells. **C.** Nascent rRNA was visualized by IF staining of incorporated 5-FU. HeLa cells were transfected with 30 nM cy3-labled ASOs and treated with DMSO or CX5461 (120 nM) for 2.5 hrs. Cells were pulsed with 1 mM 5-FU for 10 mins.

To better understand the function of NEAT1 lncRNA during cellular stress related to transcriptional inhibition of RNAP I, we depleted NEAT1 by antisense oligonucleotides (ASOs) designed to target the 5’-region shared between NEAT1 isoforms (NEAT1_1 and NEAT1_2) through a RNase H1-dependent mechanism (Fig 1A). NEAT1 ASO-1 contains 10 deoxyribonucleotides in the middle flanked at both ends by five 2’-O-methoxyethyl (2’-MOE) modified ribonucleotides, and is fully phosphorothioate (PS) modified. PS modification allows the localization of ASOs to the nucleus to ensure the effective depletion of nuclear retained NEAT1 RNA [13]. HeLa cells were either mock transfected or transfected with 15 nM ASOs (NEAT1 ASO-1 or control ASO) for 2 hrs before the treatment with RNAP I inhibitor CX5461 for another 2.5 hrs (Fig 1B). CX5461 inhibits rRNA transcription with an IC50 in the nanomolar range in different cell lines without affecting transcription from RNAP II, DNA replication, or protein translation [14, 15]. Depletion of NEAT1 lncRNA significantly alleviated the reduction of 47S rRNA precursor, as evidenced by qRT-PCR (Fig 1B) [16, 17]. We found that CX5461 does not affect the levels of NEAT1 RNA (S1A Fig). In addition, the association of NEAT1 lncRNA with paraspeckle protein p54nrb (S1A Fig) and localization of NEAT1 lncRNA in paraspeckles (S1B Fig) were not significantly affected by CX5461.

To confirm that NEAT1 depletion indeed rescued rRNA transcription instead of altering the stability or the processing of rRNA precursor, nascent RNA was labeled by brief pulsing with 5-FU for 10 mins and visualized by anti-BrdU antibody in HeLa cells transfected with either NEAT1 or control ASOs, Cy3-labeled at 5’-end (Fig 1C). Comparable nucleolar signals for nascent rRNA were observed between control ASO- or NEAT1 ASO- transfected cells without RNAP I inhibition, suggesting that NEAT1 depletion has no effect on rRNA synthesis under normal conditions. Complete loss of nucleolar but not nucleoplasm signals of 5-FU was observed in control ASO-transfected cells upon CX5461, confirming the transcriptional inhibition of RNAP I but not RNAP II by CX5461 [14]. Inhibitory effect of CX5461 on RNAP I but not RNAP II was also determined by qRT-PCR for the level of NCL1 mRNA precursor (S1C Fig). However, although not fully rescued, signals of 5-FU incorporation were observed in the nucleoli in NEAT1-depleted cells (Fig 1C). Together, our data suggest that the depletion of NEAT1 RNA moderately alleviates the CX5461-induced transcriptional inhibition of rRNA.

### CX5461-induced nucleolar disruption was significantly attenuated in NEAT1-depleted cells

**Fig 2:**
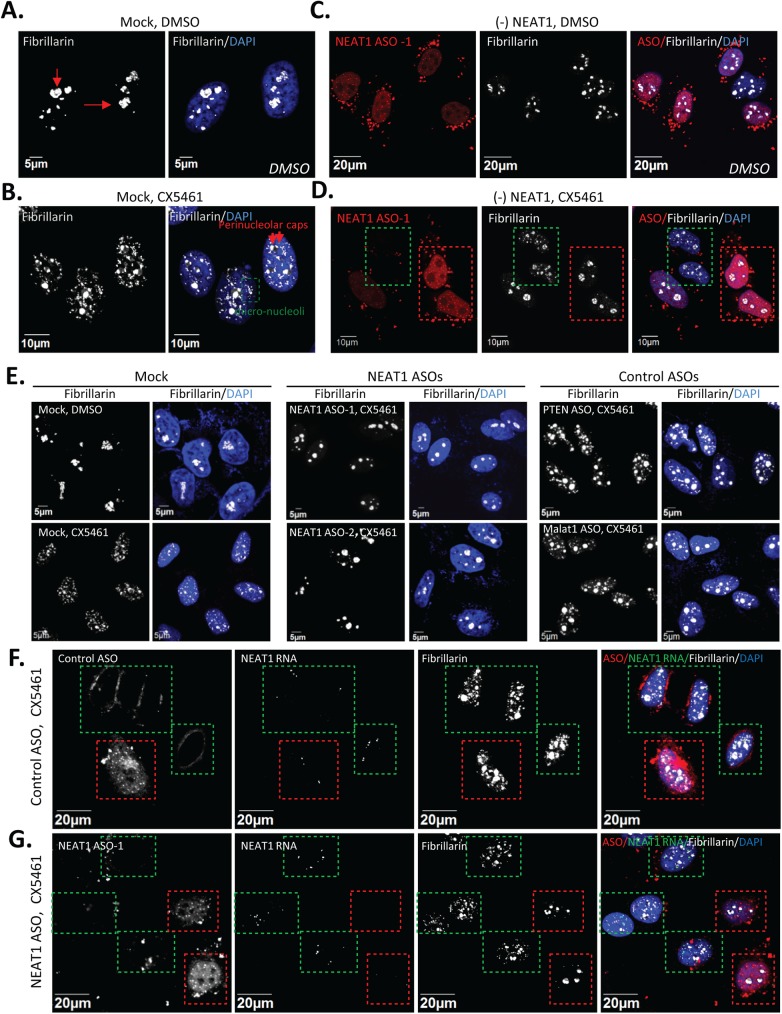
ASO-mediated depletion of NEAT1 alleviated the RNAP I inhibition induced nucleolar disruption. **A.** IF showed the localization of fibrillarin to nucleoli (red arrows) in DMSO-treated HeLa cells. **B.** Fibrillarin redistributed to nucleoplasmic foci (the green box) or perinucleolar caps (red arrows) by CX5461. **C.** NEAT1 ASOs had no effect on the distribution of fibrillarin in DMSO treated cells. Cy3-labled NEAT1 ASOs (30 nM) were transfected for 2 hrs, followed by DMSO treatment for 2.5 hrs. **D.** Redistribution of fibrillarin to nucleoplasmic foci by CX5461 was prevented in cells containing NEAT1 ASOs (the red box). HeLa cells were transfected with 30 nM NEAT1 ASOs for 2 hrs, followed by CX5461 treatment (250 nM) for 2.5 hrs. **E.** Alleviated CX5461-induced nucleolar disruption was specific for NEAT1 depletion. HeLa cells were treated as described in Fig 2D with multiple NEAT1 ASOs or control ASOs. **F and G.** Combined NEAT1-FISH and IF of fibrillarin in HeLa cells. HeLa cells were treated as described in Fig 2D with either control ASO (**F.**) or NEAT1 ASO-1 (**G.**). Cells that contain enriched amount of ASOs were boxed in red. Cell that contain little or no ASOs were boxed in green.

Inhibition of ribosomal biogenesis often results in dramatic changes in the organization and composition of the nucleolus. Delocalization of the nucleolar marker fibrillarin from nucleoli in control DMSO-treated HeLa cells (Fig 2A) to numerous small nucleoplasmic foci in CX5461-treated cells (Fig 2B) was observed (S2A Fig). These small nucleoplasmic foci appear to co-localize with coilin, a marker for Cajal bodies (CBs) (S2B Fig). The numbers of CBs per nucleus were increased from 2–3 in untreated HeLa cells to about 10 in CX5461-treated cells. A similar redistribution pattern of fibrillarin from nucleoli to nucleoplasm has been reported in Hep-2 cells treated with low concentration (0.05 mg/L) of actinomycin D that inhibits only RNAP I [18], suggesting that the formation of fibrillarin-containing nucleoplasmic foci may be a general indicator of RNAP I inhibition-related nucleolar disruption. To evaluate the effects of NEAT1 depletion on nucleolar organization under RNAP I inhibition, we transfected HeLa cells with cy3-labeled NEAT1 ASO for 2 hrs, followed by the treatment with either DMSO or CX5461 for another 2.5 hrs. NEAT1 depletion had no significant effect on the integrity of nucleoli, as suggested by the nucleolar localization of fibrillarin (Fig 2C). However, the formation of the nucleoplasmic fibrillarin foci in response to RNAP I inhibition by CX5461 was significantly reduced, if not completely prevented, in cells with NEAT1 ASOs (boxed in red), as compared with cells containing little or no ASOs (boxed in green) (Fig 2D and S2C Fig).

To excluded potential off-target hybridization effect of ASO treatment, the specificity of NEAT1-depelteion in alleviating RNAP I inhibition-induced nucleolar disruption was further validated with two additional NEAT1 ASOs of different sequence (Fig 2E and S2C Fig). Two additional control ASOs were also included, one targeting the exon region of PTEN mRNA and one targeting another highly abundant nuclear retained lncRNA Malat1. Both control ASOs showed no effect on alleviating CX5461-induced nucleoplasmic fibrillarin foci.

**Fig 3:**
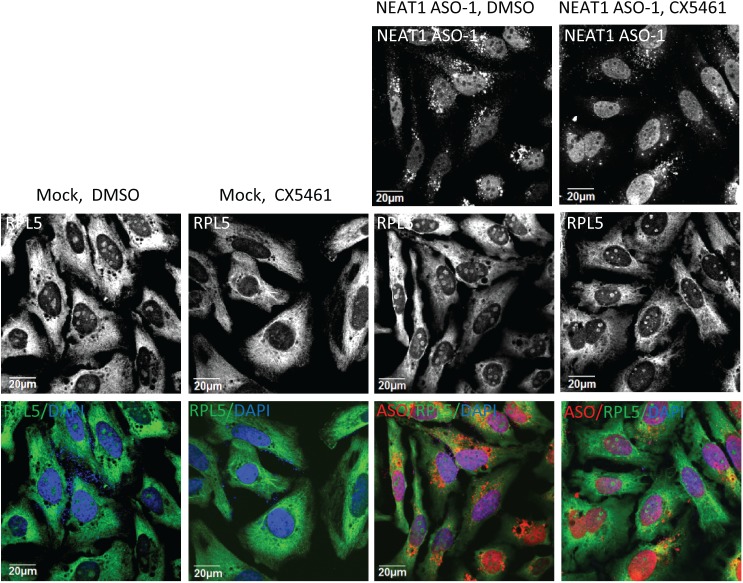
NEAT1 depletion rescued the clearance of RPL5 from nucleoli by CX5461. HeLa cells were mock-transfected or transfected with 30 nM NEAT1 ASOs, followed by the treatment with DMSO or 125 nM CX5461 for 6 hrs.

To confirm that the attenuated nucleolar disruption in NEAT1 ASO-treated cells is directly related with the levels of NEAT1 lncRNA in individual cells, sequential RNA-FISH of NEAT1 and immunofluorescence (IF) of fibrillarin were performed in cells transfected with either control ASO or NEAT1 ASO in the presence of CX5461. The FISH probe for detecting NEAT1 lncRNA was designed to base pair at the 5’-region shared between NEAT1 isoforms. In control cells, NEAT1 localize to the irregular-shaped nuclear paraspeckle foci (S1B Fig). Treatment of CX5461 did not significantly alter the subcellular distribution of NEAT1 lncRNA but caused significant redistribution of fibrillarin into numerous small nucleoplasmic foci (S1B Fig). With the transfection of control ASOs, as expected, comparable amounts of NEAT1-FISH signals as well as a similar pattern of CX5461-induced nucleoplasmic fibrillarin foci were observed in cells with (boxed in red) or without (boxed in green) ASOs (Fig 2F). For the cells transfected with NEAT1 ASO, NEAT1-FISH signals were absent in cells containing ASOs, and no nucleoplasmic fibrillarin foci were present in these NEAT1-abscent cells (boxed in red) (Fig 2G). Nucleolar disruption by CX5461 in HeLa cells also resulted in the clearance of ribosomal protein RPL5 from the nucleoli, which was also rescued by the depletion of NEAT1 lncRNA (Fig 3). Together, our results suggest that the depletion of NEAT1 lncRNA rescued CX5461-mediated nucleolar stress.

### Loss of nuclear paraspeckles is not directly responsible for coordinating cellular response to RNAP I inhibition

**Fig 4:**
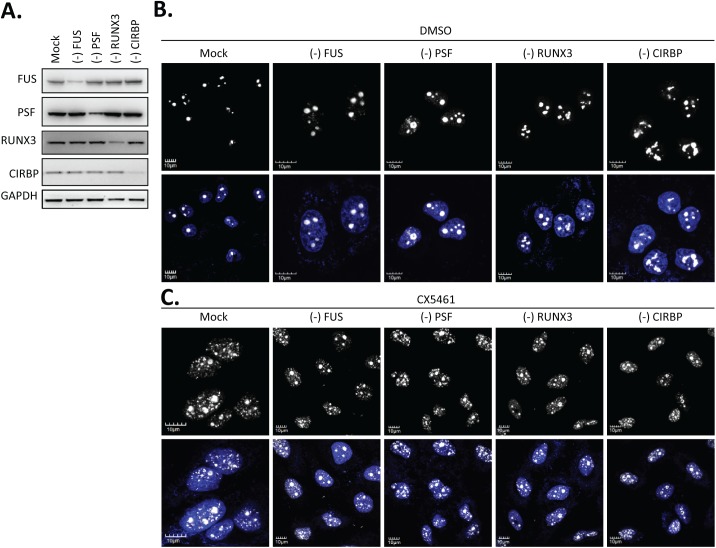
CX5461-induced nucleolar disruption was not attenuated upon disassembly of paraspeckles by depleting essential protein components. **A.** Western analysis of protein reduction by corresponding siRNAs. **B.** Depletion of the selected paraspeckle proteins has no effect on nucleolar localization of fibrillarin in the presence of DMSO. **C.** Depletion of the selected paraspeckle proteins failed to rescue the CX5461-induced mislocalization of fibrillarin to nucleoplasm.

Since the depletion of NEAT1 lncRNA resulted in the loss of paraspeckle foci, it is possible that the attenuated nucleolar stress by the NEAT1 ASO was due to the loss of paraspeckle integrity. To test this hypothesis, we examined the CX5461-mediated induction of nucleoplasmic fibrillarin-containing foci in cells depleted of individual paraspeckle-localized proteins that are either essential or non-essential for paraspeckle integrity (Fig 4A). Depletion of PSF or FUS was reported to abolish the formation of paraspeckles, while the depletion of CIRBP or RUNX3 had no effect on the size or numbers of paraspeckles [1]. Depletion of those individual proteins did not affect the nucleolar localization of fibrillarin in the absence of the RNAP I inhibitor CX5461 (Fig 4B). In the presence of CX5461, nucleoplasmic localization of fibrillarin foci were observed for cells depleted of either essential or non-essential paraspeckle proteins (Fig 4C), suggesting that the attenuated nucleolar stress in the absence of NEAT1 lncRNA is not completely due to the loss of integral paraspeckles.

### Depletion of NEAT1 during RNAP I inhibition resulted in the increased levels of c-Myc protein by releasing the NEAT1-sequestered P54nrb and PSF, positive regulators of c-Myc translation

**Fig 5:**
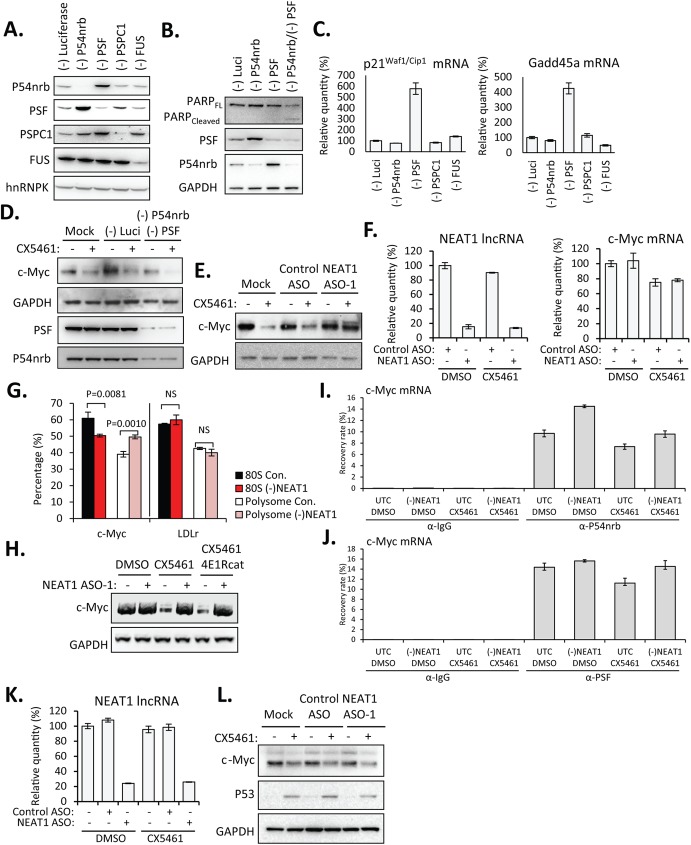
Depletion of NEAT1 lncRNA allowed increased association between P54nrb/ PSF and c-Myc mRNA to facilitate the IRES-translation of c-Myc. **A.** Western analysis of protein reduction by corresponding siRNAs. **B.** Simultaneous depletion of P54nrb and PSF by siRNAs caused proteolytic cleavage of PARP. **C.** qRT-PCR suggested the upregulation of p21^Waf1/Cip1^ and Gadd45a mRNAs upon depletion of PSF. **D.** Simultaneous depletion of P54nrb and PSF by siRNAs reduced levels of c-Myc protein in the presence of CX5461. HeLa cells were transfected with individual siRNAs at a final concentration of 3 nM for 48 hrs, followed by the treatment with DMSO or 125 nM CX5461 for 6 hrs. **E.** Depletion of NEAT1 lncRNA rescued levels of c-Myc protein in the presence of CX5461 in HeLa cells. HeLa cells were mock-transfected or transfected with 30 nM NEAT1 or control ASOs for 2hrs, followed by the treatment with DMSO or 125 nM CX5461 for 6 hrs. **F.** qRT-PCR showed levels of NEAT1 lncRNA and c-Myc mRNA in HeLa cells. **G.** qRT-PCR for relative levels of c-Myc and control LDLr mRNAs in pooled polysome (F12-F25) and mono-ribosome (80S) (F6-F10) fractions from CX5461-treated HeLa cells transfected with NEAT1 ASO-1 or control ASO (con.), as in S5 Fig). Error bars indicate s.d. of three independent experiments. P values were calculated based on unpaired t-test. NS, not significant. **H.** HeLa cells were mock-transfected or transfected with 30 nM NEAT1 for 2 hrs, followed by the treatment with DMSO, CX5461 (125 nM), or CX5461 (125 nM)/4E1Rcat (10 μM) for 6 hrs. **I and J.** qRT-PCR of levels of c-Myc RNA co-immunoprecipitated with anti-P54nrb (**I.**) or anti-PSF (**J.**) antibodies. Immunoprecipitation by anti-mouse IgG antibody serves as a background control. Results are presented as percent recovery from the input material. **K.** qRT-PCR showed levels of NEAT1 lncRNA in MCF7 cells. **L.** Depletion of NEAT1 lncRNA failed to rescue the levels of c-Myc protein in the presence of CX5461 in MCF7 cells. Experiments were performed as described in Fig 5E.

Our results suggest that it is the levels of NEAT1 RNA instead of the integral paraspeckle structures or paraspeckle number that determine the cellular response to nucleolar stress. We hypothesized that some proteins that were normally sequestered by NEAT1 via direct association might be released upon NEAT1 depletion to help cells cope with stress. Indeed, the levels of functional paraspeckle proteins P54nrb and PSF were found to correlate positively with the cell survival [19]. In HeLa cells, depletion of either P54nrb or PSF resulted in the compensatory upregulation of the other paraspeckle proteins (Fig 5A), consistent with the previous observation in P54nrb knockout animals and in patients with mutations in P54nrb [20–22]. P54nrb, PSF, and PSPC1 all contain the same DBHS domain (for Drosophila behavior, human splicing). It has been reported that P54nrb deficient MEF cells were only mildly radiosensitive, which was attributable to the upregulation of PSPC1, but cells lacking both P54nrb and PSPC1 were markedly radiosensitive and deficient in double strand DNA damage repair [20]. In addition to DNA repair, DBHS proteins were also reported to be involved in many cellular processes including RNA transcription, splicing, 3’-end processing, and translation [23]. Importantly, apoptosis-specific 89 kDa PARP fragment (cleaved-PARP) was detected upon simultaneous depletion of both P54nrb and PSF in HeLa cells (Fig 5B). Moreover, siRNA-mediated depletion of PSF, but not p54nrb, PSPC1, or FUS in Hela cells induced significant upregulation of mRNAs transcriptionally downstream of p53, including p21^Waf1/Cip1^ and Gadd45a (Fig 5C).

Consistent with the observed positive roles of P54nrb and PSF in cell proliferation, it has been reported that both proteins bind directly to c-Myc mRNA and are required for the IRES-dependent translation of c-Myc [10]. Since c-Myc is a master regulator of ribosomal biogenesis (S3 Fig), it is not surprising that the protein levels of c-Myc influence cellular response to nucleolar stress. RNAP I inhibition in HeLa cells by CX5461 caused a reduction in protein levels of c-Myc, but further loss of c-Myc protein was observed in cells depleted of both P54nrb and PSF simultaneously (Fig 5D), consistent with their function in enhancing c-Myc translation. Importantly, depletion of NEAT1 rescued the reduction in the levels of c-Myc protein during RNAP I inhibition (Fig 5E), which may at least partially explain the attenuated transcriptional inhibition and nucleolar disruption by CX5461 in NEAT1-absent cells. Moderately alleviated cell death in the presence of CX5641 was observed in NEAT1-depleted cells, which was at least partially related with the attenuated reduction of c-Myc protein (S4 Fig). We noticed that the NEAT1-depletion has no significant effect on the levels of c-Myc protein under normal growth conditions, implying that NEAT1-depletion may increase the levels of c-Myc protein through a mechanism that active during cellular stress.

Increased levels of c-Myc protein in the absence of NEAT1 did not occur at RNA level. Levels of c-Myc mRNA was not significantly affected, if not slightly reduced, by CX5461 (Fig 5F). However, depletion of NEAT1 lncRNA in the presence of CX5461 shifted c-Myc mRNA, but not the control LDLr mRNA, towards polysome, as suggested by polysome profile assay (S5 Fig). An increased ratio of c-Myc mRNA in polysome vs. mono-ribosome fractions was observed (Fig 5G), suggesting that during RNAP I inhibition, translation of c-Myc was increased in cells depleted of NEAT1 lncRNA. Interestingly, inhibition of cap-dependent translation by a small molecule inhibitor 4E1Rcat did not abolish the effect of NEAT1 depletion on attenuating CX5461-induced reduction in protein levels of c-Myc (Fig 5H), suggesting that cap-dependent translation was not responsible for the increased levels of c-Myc protein.

Delocalization of PSF from the nucleus to the cytoplasm during apoptosis was observed previously, correlating with the role of PSF in mediating IRES translation of many proteins. To test the hypothesis that the depletion of NEAT1 lncRNA can facilitate IRES translation of c-Myc by redistributing P54nrb and PSF from nuclear paraspeckles onto the c-Myc mRNA, we examined the association of P54nrb or PSF with c-Myc mRNA by RNA binding protein immunoprecipitation (RNA-IP) (Fig 5I and 5J). An increased fraction of c-Myc mRNA was found to be bound either by P54nrb (Fig 5I) or PSF (Fig 5J) in NEAT1-depleted cells, suggesting that NEAT1 depletion releases its sequestered P54nrb and PSF.

IRES-dependent translation of c-Myc varies between cells lines. While active in HeLa cells, c-Myc IRES is inactive in MCF7 cells [24], which enabled us to analyze whether NEAT1-depletion-induced increasing in levels of c-Myc protein was mediated by IRES translation. Similar to the observation in HeLa cells, reduced levels of c-Myc protein were observed in MCF7 cells with the treatment of CX5461. However, the depletion of NEAT1 lncRNA failed to rescue the levels of c-Myc protein in MCF7 cells (Fig 5K and 5L), implying that the increased levels of c-Myc protein in NEAT1-depleted HeLa cells were at least partially due to the IRES-translation of c-Myc under stress conditions.

## Discussion

Numbers and morphology of paraspeckles, as well as the distribution and abundance of paraspeckle components, both proteins and RNAs, are altered rapidly in response to changes in cellular conditions, including differentiation, DNA damage, transcriptional inhibition, hypoxia, viral infection, immune stimuli, apoptosis, and oncogenic stress. Being the initial seeding molecule of paraspeckles, levels of NEAT1 directly determine the size and morphology of paraspeckles as well as the subcellular distribution of proteins associated with NEAT1. Many paraspeckle proteins, P54nrb and PSF, in particular, are essential proteins for multiple cellular processes, including transcription, splicing, polyadenylation, nuclear retention, translation, and DNA repair [23]. P54nrb, PSF, along with the third paraspeckle protein PSPC1, belong to the same family termed DBHS (Drosophila behavior human splicing) based on sequence homology. Levels of individual DBHS proteins compensate each other [20, 21], suggesting that levels of these proteins need to be maintained to allow proper cellular functions. Indeed, we showed that simultaneous depletion of P54nrb and PSF caused apoptosis in HeLa cells. In addition, PSF contains N-terminal RGG boxes and proline-rich sequence which are not included in other family members. Consequently, PSF has additional functions not ascribed to P54nrb and PSPC1, which explains the upregulated p21^Waf1/Cip1^ and Gadd45a mRNAs in HeLa cells depleted of PSF but not P54nrb or PSPC1. This observation is also consistent with the previous reports that PSF, but not P54nrb or PSPC1, is critical for the survival of zebrafish and murine T cells [19, 25].

Upregulation of NEAT1 during apoptosis seems to be in agreement with the correlation between P54nrb/PSF and cell survival. It is possible that P54nrb and PSF are recruited and sequestered by the upregulated NEAT1 in nuclear foci to further facilitate apoptosis. Of course, sequestration of other paraspeckle proteins that are involved in apoptosis or proliferation, such as the anti-apoptosis protein XIAP, by NEAT1 may also contribute to the cell death. Considering the function of P54nrb and PSF in activating IRES-translation of c-Myc, reduction or sequestration of P54nrb and PSF will certainly reduce cell proliferation. We do not exclude the possibility that P54nrb and PSF may contribute to the cell survival through mechanisms other than activating c-Myc translation. In fact, P54nrb has been reported as a transcriptional activator for anti-apoptotic protein Survivin [26].

IRES-translation of c-Myc varies between cell lines. Among cell lines tested previously, IRES-translation of c-Myc is most robust in HeLa cells but almost inactive in MCF7 [24]. It is possible that MCF7 lacks the expression of essential factors activating c-Myc translation. This hypothesis is supported by the observation that although P54nrb and PSF are expressed in MCF7, localization of P54nrb and PSF is predominantly in the perinucleolar regions instead of paraspeckles in some cells [27]. MCF7 lacks enzymes (*e*.*g*. MTAP) to maintain the methylation status of paraspeckle proteins such as FUS, leading to the altered distribution of hypo-methylated FUS and its associated P54nrb and PSF to the perinucleolar space [27]. Mislocalization of P54nrb and PSF may affect their interaction with NEAT1 and/or their dynamics such as nucleo-cytoplasmic shuttling, reducing their association with c-Myc mRNA and thus rendering c-Myc IRES inactive in MCF7.

c-Myc has been reported to modulate ribosomal biogenesis, especially RNAP I transcription, in multiple ways. For example, c-Myc can serve as a transcription activator for core RNAP I subunits including UBF [28]. c-Myc can also associate with SL1 to stabilize SL1/UBF interaction during transcription initiation of RNAP I [29]. Moreover, c-Myc can bind directly to rDNA and recruit RNAP I [29]. CX5461 inhibits rRNA synthesis by acting as a competitive inhibitor for SL1 [15], preventing the association of SL1 with rDNA promoter. It is possible that the increased protein levels of c-Myc rescued the rRNA synthesis by competing with CX5461 for SL1 binding, although the detailed mechanisms require further evaluation.

We published previously that PS-modified ASOs of several of 2’-modifications can interact with proteins in paraspeckles, such as P54nrb, PSF, PSPC1, hnRNPK, FUS and TDP43, in an ASO dose-dependent manner [11, 22]. The interaction between PS-ASOs with these proteins leads to the localization of PS-ASOs in paraspeckles. These observations may complicate the interpretation of results acquired from NEAT1 depletion by antisense mechanism. To avoid the possible secondary effects due to the ASO-P54nrb/PSF interaction, ASO-mediated depletion of NEAT1 in this study was performed with lowest ASO concentration possible and included multiple NEAT1 ASOs and control ASOs in the experiments. The dose-dependent reduction of NEAT1 RNA as well as the localization of NEAT1 ASO and P54nrb were carefully monitored. The maximum reduction of NEAT1 RNA was achieved by 15 nM ASOs, while the co-localization of PS-ASO and P54nrb was only observed at concentrations above 45 nM (S6 Fig). In addition, multiple NEAT1 ASOs and control ASOs were examined and included to validate that the observed phenotypes are not due to the potential off-target cleavages by ASOs.

Sequestration of multi-functional proteins P54nrb and PSF by non-coding transcripts, including NEAT1, Malat1 and mouse VL30-1, or coding transcripts, such as MER11C and L1PA16, was reported previously [30], leading to altered cellular processes. Here we provide a further example showing that the availability of functional P54nrb and PSF to activate IRES-dependent translation of c-Myc during stress conditions is regulated by the levels of NEAT1 lncRNA. Considering the positive correlation between the levels of P54nrb/PSF and cell survival, we propose that NEAT1 RNA may have a role in fine tuning the availability of these proteins to allow the balance between proliferation and apoptosis.

## Supporting information

S1 Fig Abundance and localization of NEAT1 RNA was not affected by the treatment with CX5461. **A.** qRT-PCR showed levels of NEAT1 lncRNA in HeLa cells in the presence of DMSO or CX5461. RNA-IP showed that the association of NEAT1 lncRNA with P54nrb was not significantly affected by CX5461. **B.** HeLa cells were treated as described in Fig 2A and 2B. Combined NEAT1-FISH and IF staining of fibrillarin was performed. **C.** Treatment with CX5461 did not significantly affect the levels of RNAP II transcripts, as exemplified by the levels of NCL1 mRNA precursor quantified using qRT-PCR assay.
(TIF)

S2 Fig Depletion of NEAT1 attenuated the nucleolar stress induced by CX5461-treatment. **A.** Population view of fibrillarin localization in DMSO or CX5461-treated cells. **B.** Co-localization (marked by yellow arrows) of fibrillarin containing nucleoplasmic foci with Cajal body marker coilin. **C.** Two NEAT1 ASOs of difference sequence both effectively attenuated the mislocalization of fibrillarin into numerous nucleoplasmic foci.
(TIF)

S3 Fig Depletion of c-Myc significantly reduced rRNA synthesis. HeLa cells were either mock-transfected or transfected with c-Myc siRNA for 24 hrs before pulsed with 1 mM 5-FU for 10 mins. Nascent RNA were visualized by immunofluorescence staining using anti-BrdU antibody for incorporated 5-FU.
(TIF)

S4 Fig Depletion of NEAT1 lncRNA attenuated cell death induced by CX5461. WST-8 assay suggested that CX5461-induced cell death was modestly attenuated upon the depletion of NEAT1 lncRNA. The error bars are standard deviation of three experiments.
(TIF)

S5 Fig c-Myc mRNA shifted towards the polysome fractions in CX5461-treated HeLa cells depleted of NEAT1 lncRNA. RNA was prepared from different fractions and qRT-PCR was performed using primer probe sets specific to 28S rRNA (28S), c-Myc mRNA, and LDLr mRNA. The percentages of each fraction are plotted. The error bars are standard deviation of 3 experiments. Polysome fractions: F12-F25 and mono-ribosome (80S): F6-F10.
(TIF)

S6 Fig Dose-dependent reduction in NEAT1 RNA levels by NEAT1 ASOs. **A.** Dose-dependent reduction of NEAT lncRNA by NEAT1 ASO-1. NEAT1 ASOs were transfected to HeLa cells at the specified concentration for 2 hrs. Levels of NEAT1 lncRNA were determined by qRT-PCR. The error bars represent standard deviations from three experiments. **B.** IF staining of P54nrb in HeLa cells transfected with NEAT1 ASO-1 at the specified concentration. Localization of P54nrb to the paraspeckles was observed in the absence of NEAT1 ASOs. NEAT1 levels were significantly reduced by as low as 7.5 nM NEAT1 ASOs to prevent the formation of paraspeckle. Co-localization of PS-ASOs and P54nrb was only observed when ASOs were transfected at high concentration (45 nM).
(TIF)

S1 Text Supplemental Materials and Methods. (PDF)
