# Supplementary figures and images for: Depletion of NEAT1 lncRNA attenuates nucleolar stress by releasing sequestered P54nrb and PSF to facilitate c-Myc translation
Source: PLoS One. 2017 Mar 13;12(3):e0173494. doi: 10.1371/journal.pone.0173494 (PMC5348036; doi:10.1371/journal.pone.0173494)

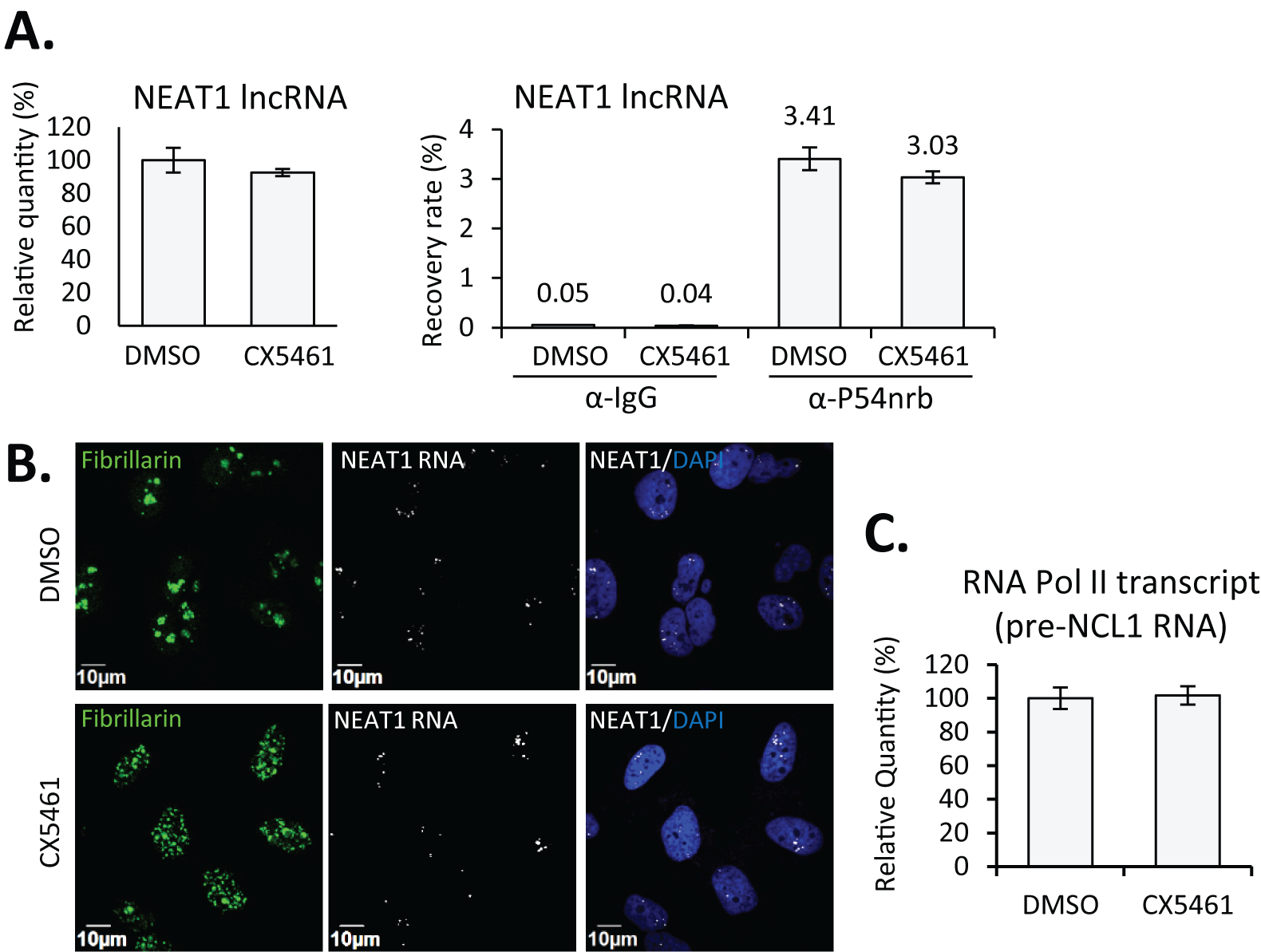

Supplement: S1 Fig — A. qRT-PCR showed levels of NEAT1 lncRNA in HeLa cells in the presence of DMSO or CX5461. RNA-IP showed that the association of NEAT1 lncRNA with P54nrb was not significantly affected by CX5461. B. HeLa cells were treated as described in Fig 2A and 2B. Combined NEAT1-FISH and IF staining of fibrillarin was performed. C. Treatment with CX5461 did not significantly affect the levels of RNAP II transcripts, as exemplified by the levels of NCL1 mRNA precursor quantified using qRT-PCR assay. (TIF) [file pone.0173494.s001.tif]

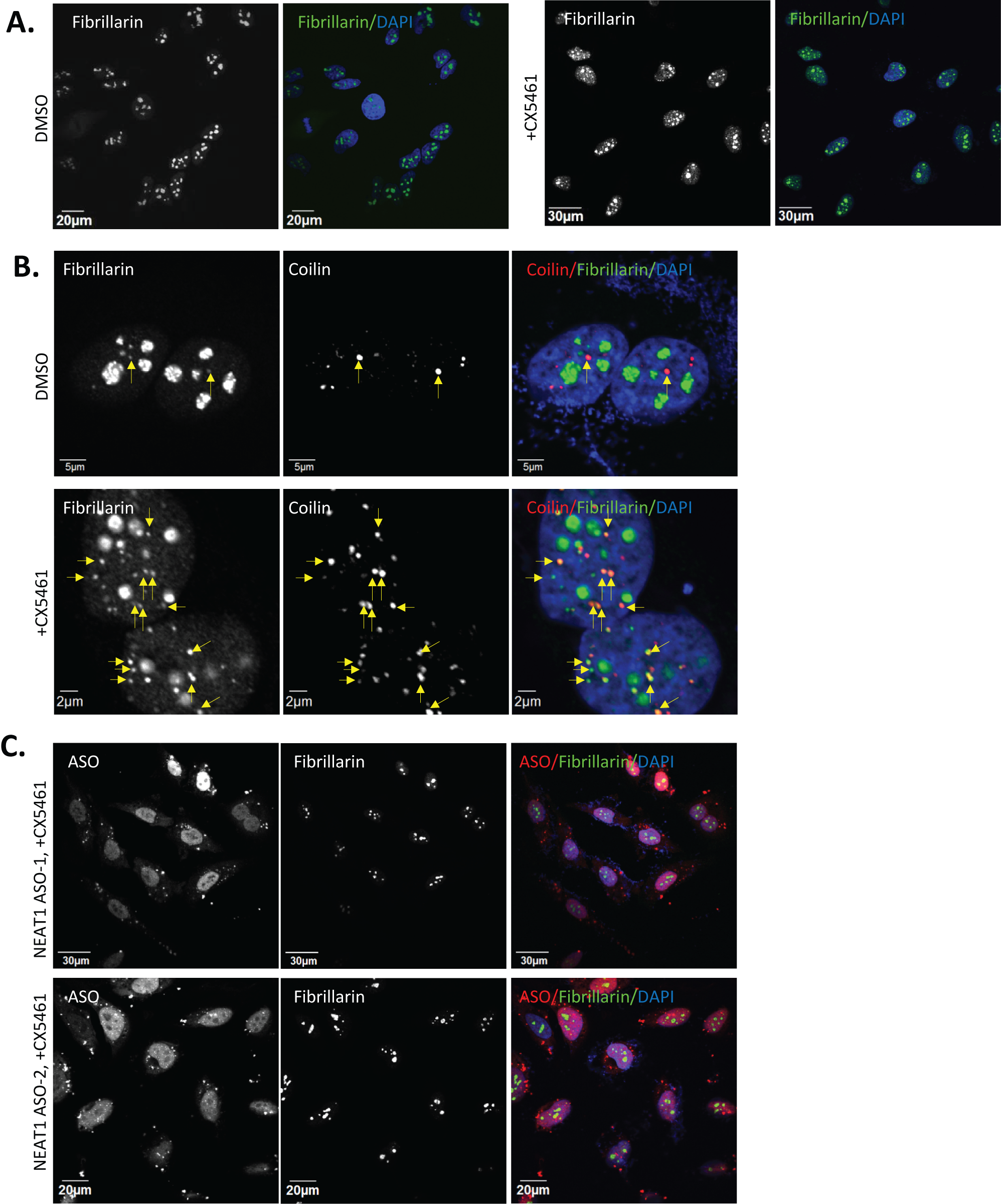

Supplement: S2 Fig — A. Population view of fibrillarin localization in DMSO or CX5461-treated cells. B. Co-localization (marked by yellow arrows) of fibrillarin containing nucleoplasmic foci with Cajal body marker coilin. C. Two NEAT1 ASOs of difference sequence both effectively attenuated the mislocalization of fibrillarin into numerous nucleoplasmic foci. (TIF) [file pone.0173494.s002.tif]

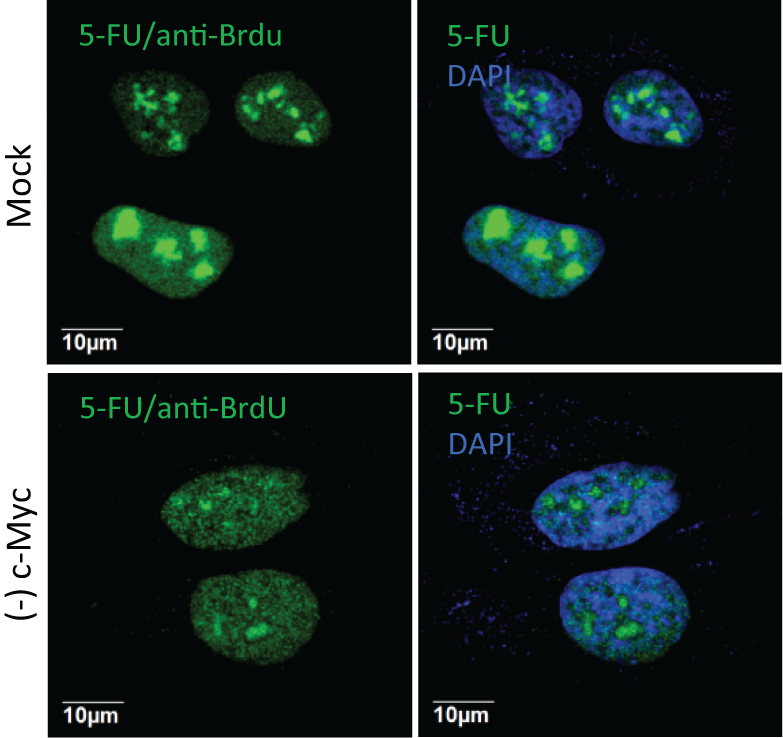

Supplement: S3 Fig — HeLa cells were either mock-transfected or transfected with c-Myc siRNA for 24 hrs before pulsed with 1 mM 5-FU for 10 mins. Nascent RNA were visualized by immunofluorescence staining using anti-BrdU antibody for incorporated 5-FU. (TIF) [file pone.0173494.s003.tif]

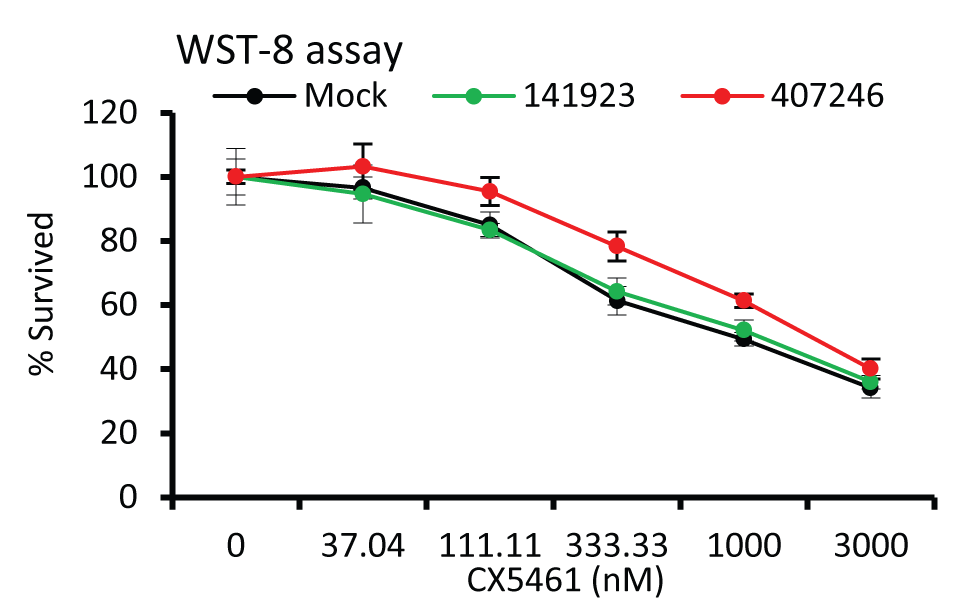

Supplement: S4 Fig — WST-8 assay suggested that CX5461-induced cell death was modestly attenuated upon the depletion of NEAT1 lncRNA. The error bars are standard deviation of three experiments. (TIF) [file pone.0173494.s004.tif]

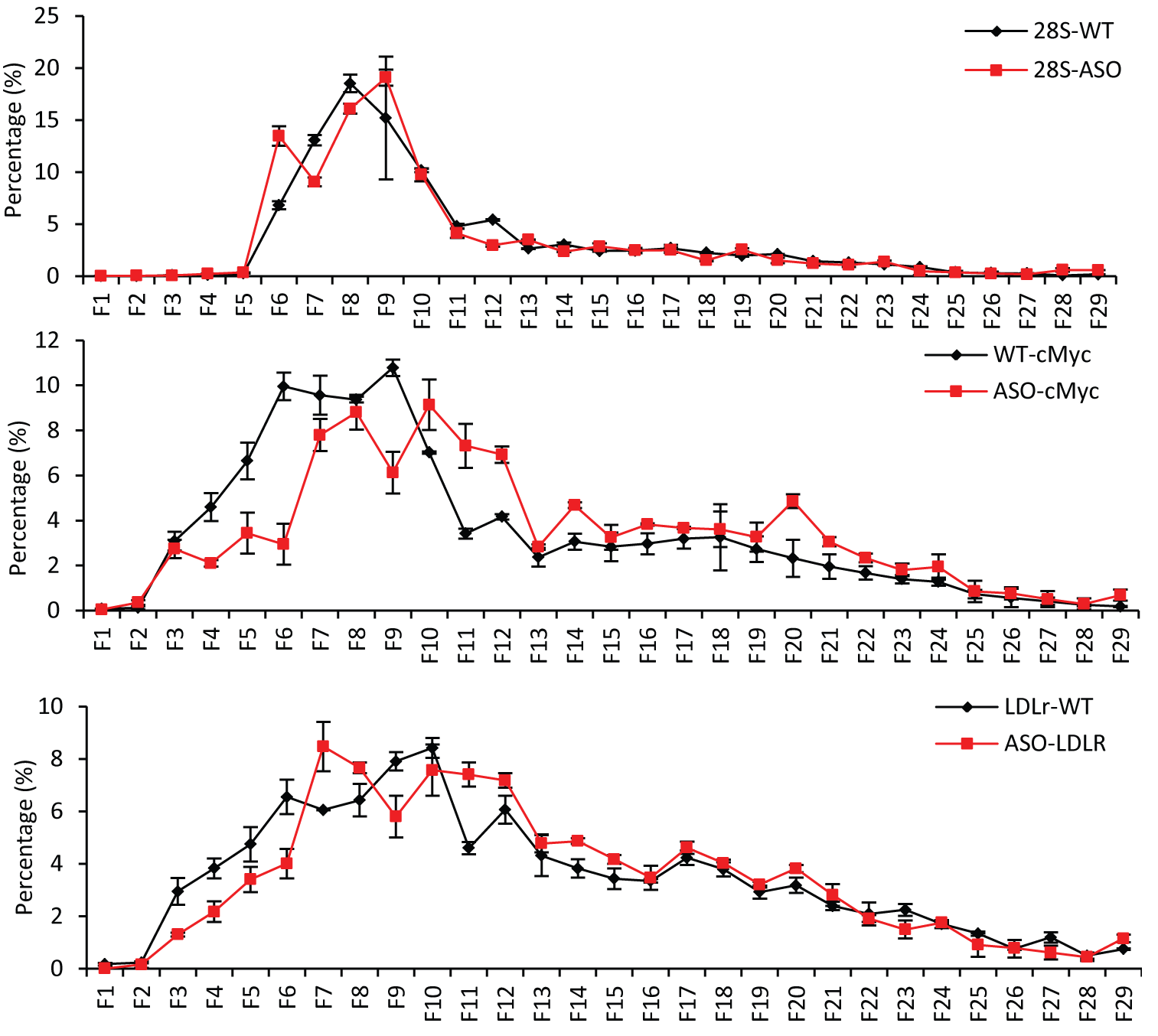

Supplement: S5 Fig — RNA was prepared from different fractions and qRT-PCR was performed using primer probe sets specific to 28S rRNA (28S), c-Myc mRNA, and LDLr mRNA. The percentages of each fraction are plotted. The error bars are standard deviation of 3 experiments. Polysome fractions: F12-F25 and mono-ribosome (80S): F6-F10. (TIF) [file pone.0173494.s005.tif]

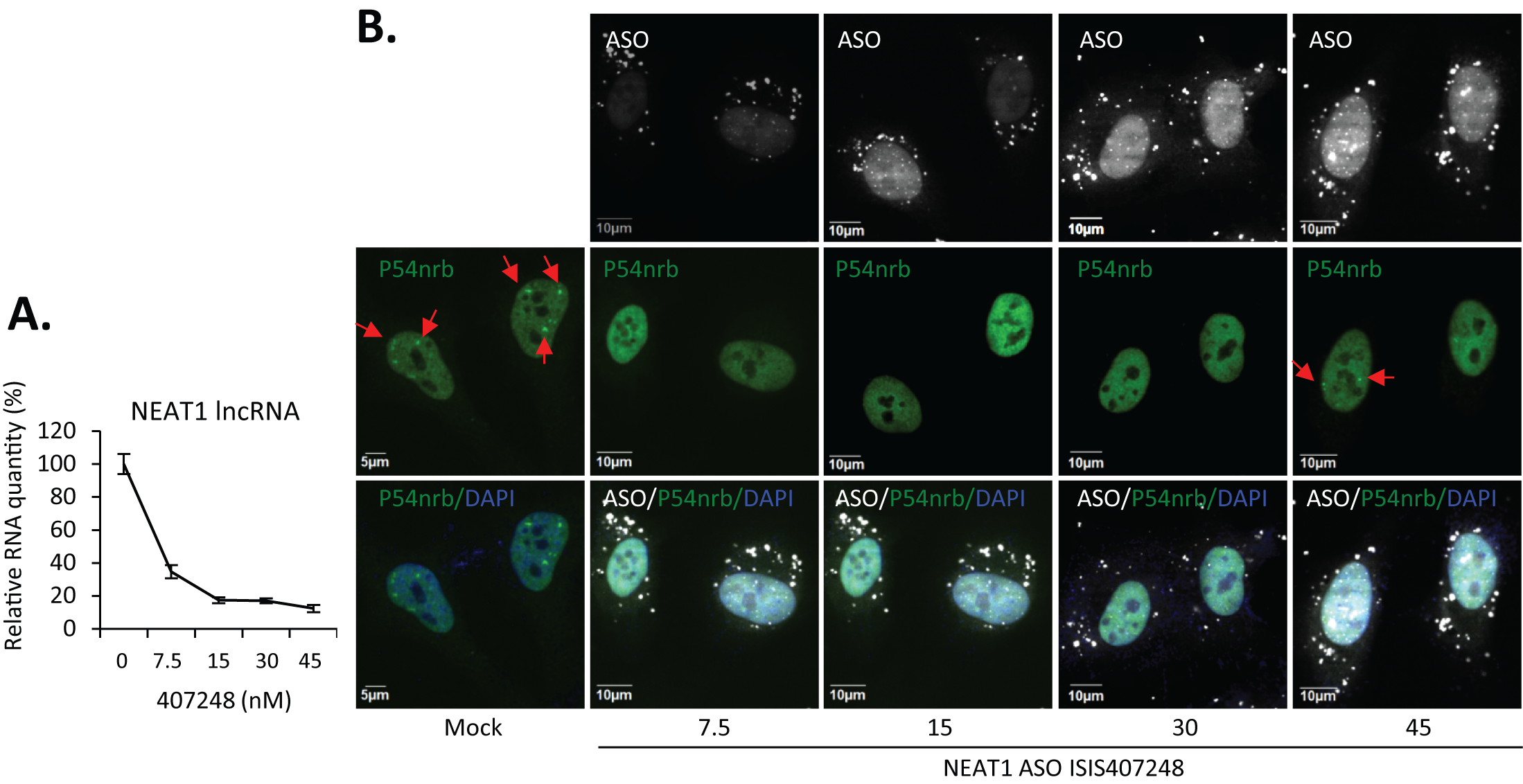

Supplement: S6 Fig — A. Dose-dependent reduction of NEAT lncRNA by NEAT1 ASO-1. NEAT1 ASOs were transfected to HeLa cells at the specified concentration for 2 hrs. Levels of NEAT1 lncRNA were determined by qRT-PCR. The error bars represent standard deviations from three experiments. B. IF staining of P54nrb in HeLa cells transfected with NEAT1 ASO-1 at the specified concentration. Localization of P54nrb to the paraspeckles was observed in the absence of NEAT1 ASOs. NEAT1 levels were significantly reduced by as low as 7.5 nM NEAT1 ASOs to prevent the formation of paraspeckle. Co-localization of PS-ASOs and P54nrb was only observed when ASOs were transfected at high concentration (45 nM). (TIF) [file pone.0173494.s006.tif]
