# Supplementary material for: Depletion of NEAT1 lncRNA attenuates nucleolar stress by releasing sequestered P54nrb and PSF to facilitate c-Myc translation
Source: PLoS One. 2017 Mar 13;12(3):e0173494. doi: 10.1371/journal.pone.0173494 (PMC5348036; doi:10.1371/journal.pone.0173494)
Supplement: S1 Text — (PDF) [file pone.0173494.s007.pdf]

Supplemental Materials and Methods

Antibodies:

For western blotting, anti-P54nrb (sc-376865), anti-hnRNPK (sc-28380), and anti-GAPDH (sc-32233) antibodies were purchased from Santa Cruz Biotechnology. Anti-FUS (ab84078), anti-PSPC1 (ab104238), anti-CIRBP (ab191885), anti-RUNX3 (ab135248), and anti-p53 (ab28) were purchased from Abcam. Anti-c-Myc (9402) antibody was purchased from cell signaling. Anti-PARP (4338-MC-50) antibody was purchased from TREVIGEN.

For IF, anti-Fibrillarin antibody (ab5821), anti-RPL5 (ab186857), and anti-BrdU (ab6326) antibodies were purchased from Abcam. Anti-P54nrb antibody (sc-376865) was purchased from Santa Cruz Biotechnology. Anti-PSF antibody (p2860) was purchased from Sigma-Aldrich.

For RNA-IP, anti-P54nrb antibody (05-950) was purchased from Millipore, and anti-PSF antibody (p2860) was purchased from Sigma-Aldrich.

Primer and probe sequences:

The primers for NEAT1 RNA-FISH probe sub-cloning are: 5´-hNEAT1-Forward: 5´- TAG TTG TGG GGG AGG AAG TG -3´ and 5´-hNEAT1-Reverse: 5´- *TAA TAC GAC TCA CTA TAG GG*T GGC ATG GAC AAG TTG AAG A -3´. Underlined italics indicate T7 promoter sequence.

The primer-probe sets for qRT-PCR are: NEAT1-Forward: 5´- TGG TAA GCC CGG GAC AGT AA -3´; NEAT1-Reverse: 5´- CAG CGG GAA GGC CTC TCT -3´; NEAT1-Probe: 5´- CCG AGT GGC TGT TGG AGT CGG TAT TG -3´; c-Myc-Forward: 5´- TGG TCT TCC CCT ACC

CTC TCA -3´; c-Myc-Reverse: 5´- AGA ATC CGA GGA CGG AGA GAA -3´; c-Myc-Probe: 5´- CGA CAG CAG CTC GCC CAA GTC C -3´; Pre-NCL1-Forward: 5´- CTC TGT CAC TGG TAT CTT TTC CC -3´; pre-NCL1-Reverse: 5´- CAA AAC CAA ACC TAG AAC ACC AAA TG -3´; Pre-NCL1-Probe: 5´- CAA GGC TAC TTT CTG TGG GAT GGC T -3´; LDLr-Forward: 5´- CAT TCA CCA AAT GAT GCC ACT T -3´; LDLr-Reverse: 5´- CGG GTG TCT CAG GCA CTT AAT AA -3´; LDLr-Probe: 5´- AGA GCC TGA GTC ACC GGT CAC CCT TAA -3´; 28S-Forward: 5´- CAG GTC TCC AAG GTG AAC AG -3´, 28s-Reverse: 5´- CTT AGA GCC AAT CCT TAT CCC G -3´, 28S-Probe: 5´- TCC CTT ACC TAC ATT GTT CCA ACA TGC C -3´; 47S rRNA precursor-Forward: 5´- TGT CAG GCG TTC TCG TCT C -3´; and 47S rRNA precursor-Reverse: 5´-AGCACGACGTCACCACATC -3´. Primer probe sets for $p21^{Waf1/Cip1}$ and Gadd45a were purchased from Thermo Fisher Scientific.

siRNAs:

PSF siRNA-sense: 5´-CAG UCA UUG UGG AAC CAC UUG AAC A-5´, and anti-sense 5´-UGU UCA AGU GGU UCC ACA AUG ACU G-3´; FUS siRNA-sense: 5´- CGG GAC AGC CCA UGA UUA AUU UGU A -5´, and anti-sense 5´-UAC AAA UUA AUC AUG GGC UGU CCC G -3´; RUNX3 siRNA-sense: 5´- CCC UGA CCA UCA CUG UGU UCA CCA A -5´, and anti-sense 5´- UUG GUG AAC ACA GUG AUG GUC AGG G -3´; CIRBP siRNA-sense: 5´-GGA GGC UCC AGA GAC UAC UAU AGC A-5´, and anti-sense 5´-UGC UAU AGU AGU CUC UGG AGC CUC C -3´. siRNA for P54nrb (s9612 and s9614) and PSPC1 (s30594) were purchased from Life Technologies.

ASOs:

ASOs used in this study all are 5-10-5 gapmer design with10 deoxyribonucleotides in the middle flanked at both ends by five 2'-O-methoxyethyl (2'-MOE) modified ribonucleotides, and is fully phosphorothioate (PS) modified. NEAT1 ASO-1: 5′-ATG GGC TCT GGA ACA AGC AT-3′; NEAT1 ASO-2: 5′- CCA TAA GCT CAT CTG CAA AC-3′; Control ASO-1 (non-target): 5′- CCT TCC CTG AAG GTT CCT CC-3′; Control ASO-2 (Pten): 5′- CTG CTA GCC TCT GGA TTT GA-3′; Control ASO-3 (Malat1): 5′- TGC CTT TAG GAT TCT AGA CA-3′.

RNA-FISH and IF:

Cells seeded on glass-bottom culture dishes (MatTek) were fixed at room temperature for 30 min with 4% formaldehyde in PBS and permeabilized for 5 min with 0.1% Triton X-100 in PBS. For NEAT1 FISH, fluorescently labeled RNA probes were generated using FISH Tag™ RNA Multicolor Kit (Thermo Fisher Scientific) according to the manufacturer's instructions. After washing twice with 1×PBS and once with 2×saline-sodium citrate (SSC), permeabilized cells were incubated for overnight at 55 °C with RNA probes in hybridization solution (50% formamide, 2×SSC, 5% dextran sulfate, 0.5 M EDTA, 0.01% Tween-20, 100 μg/mL yeast tRNA , and 1X Denhardt's salt). Cells were washed twice in wash buffer (0.2× SSC and 50% formamide) at 55 °C for 30 min each, and mounted using Prolong Gold anti-fade reagent with DAPI (Molecular Probes). For indirect IF staining, fixed and permeabilized cells were blocked in blocking buffer (1 mg/ml BSA in PBS) at room temperature for 30 mins, and incubated with primary antibodies in blocking buffer at room temperature for 2 hrs. After washing three times in washing buffer (0.1% Tween-20 in PBS) for 5 mins each, cells were incubated with secondary antibodies in blocking buffer at room temperature for 1 hrs. Finally, cells were washed three times in washing buffer for 5 mins each and mounted using Prolong Gold anti-fade reagent with

DAPI (Molecular Probes). Images were generated by Confocal Laser Scanning Biological Microscopy FV1000 Fluoview (Olympus) and analyzed using Fluoview Ver. 2.0b Viewer (Olympus).

WST-8 assay

WST-8 assay was performed using Cell Counting Kit – 8 (Sigma) according to manufacturer's instructions.
